# Walking Distance Estimation Using Walking Canes with Inertial Sensors

**DOI:** 10.3390/s18010230

**Published:** 2018-01-15

**Authors:** Duc Cong Dang, Young Soo Suh

**Affiliations:** Electrical Engineering Department, University of Ulsan, Ulsan 44610, Korea; congdd.ac@gmail.com

**Keywords:** mobility aids, walking cane, walking distance estimation, inertial sensor, Kalman filter

## Abstract

A walking distance estimation algorithm for cane users is proposed using an inertial sensor unit attached to various positions on the cane. A standard inertial navigation algorithm using an indirect Kalman filter was applied to update the velocity and position of the cane during movement. For quadripod canes, a standard zero-velocity measurement-updating method is proposed. For standard canes, a velocity-updating method based on an inverted pendulum model is proposed. The proposed algorithms were verified by three walking experiments with two different types of canes and different positions of the sensor module.

## 1. Introduction

Mobility aids are devices designed to alleviate the impact of mobility limitations or improve independence and reduce the burden of care [1,2,3,4]. They are mainly simple mechanical devices—such as wheelchairs, canes, crutches, and walkers—which enable freedom of movement similar to that of unassisted walking or standing up from a chair [2]. These assistive devices are effective ways to lessen the impact of mobility limitation for many people, including older adults, people with chronic conditions, the disabled, the blind, and people with mobility impairments. Patients using mobility aids have reported improved confidence and feelings of safety, resulting in increased activity levels and independence [3,4]. Studies have evaluated the impact of specific assistive devices on mobility outcomes and fall prevention [5].

Because the world’s elderly population is growing dramatically, the use of assistive devices is becoming increasingly important [6,7]. Currently, an estimated 6.1 million community-dwelling adults use mobility devices, including canes, walkers, and crutches, and two-thirds of them are older than 65 years. Among this group, 10 percent use canes and 4.6 percent use walkers [7].

The use of walking aids may affect the gait pattern considerably. Some people choose to use walking aids to make it easier to walk, which can reduce the pain in painful joints, while others are completely unable to walk without some form of aid [8]. Walking assistive devices can be classified into canes, crutches, and walkers, which all operate by supporting part of the body weight through the arms rather than the legs. The simplest form of walking aid is the cane, which helps redistribute body weight by transmitting force to the ground through the wrist and hand. It also improves stability by increasing the base of support, and provides tactile information about the ground [8,9]. A crutch has two points of attachment, one at the hand and one higher up the arm, so it is able to transmit significant forces in the horizontal plane [8]. Therefore, crutches are helpful for those who need to use their arms for weight bearing and propulsion and not just for balance [7]. The most stable walking aid is the walker, also called a walking frame, which improves stability for those with weakness in the lower extremity or poor balance. The user is able to stand and walk within the area of support provided by the walker’s base. However, walkers can be difficult to maneuver and can result in poor back posture and reduced arm swing [4,8,9].

Recently, there has been a substantial amount of literature aimed at classifying human activity through the use of sensory information such as acceleration and angular velocity [10]. Some of the works focus specifically on detecting fall events [11,12,13], and others investigate fall detection using walking aids [14,15,16]. Lan et al. [14] introduced the SmartFall system for the SmartCane [17,18]. A modified standard cane with a wireless module attached to the shaft collects inertial measurements and force signals. The fall detection algorithm is based on recognizing a fall pattern in three different stages: collapse, impact, and inactivity. Culmer et al. [19] developed the Instrumented Walking Aid (iWA) for the purpose of clinical evaluation. It includes a custom load cell for measuring load applied by the user and a wireless module to collect inertial information. They focused on tracking the cane’s orientation and applied load patterns.

In our work, the major focus is to assess the acceleration and angular velocity of a cane to provide precise estimation of the user’s walking distance, which is useful for caregivers or doctors to monitor the usage of the cane or the user’s rehabilitation level. We also investigate the algorithm’s performance with different types of canes and different positions of the sensor module on the cane. Several walking experiments with two types of canes were performed to evaluate the proposed algorithm.

## 2. System Description

**Figure 1:**
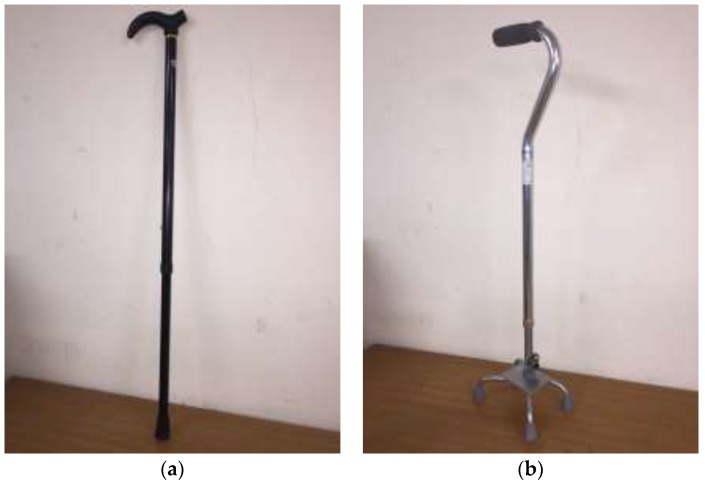
Two basic types of cane: (**a**) Standard cane; (**b**) Quadripod cane.

Several types of canes are available in many shapes and sizes, and we investigated two types: a standard cane and a quadripod cane (or quad cane) [9] (see Figure 1).

A standard cane or straight cane is generally made from inexpensive and lightweight materials such as wood or aluminum. The height of an aluminum cane can be adjusted. A standard cane can help with the balance for someone who does not need strong weight-bearing support [20].

A quadripod cane is a four-legged cane that can stand freely on its own. It provides a larger base of support and provides more weight bearing through the upper extremities. However, all four tips of the cane must be in contact with the ground at the same time for proper use [20].

**Figure 2:**
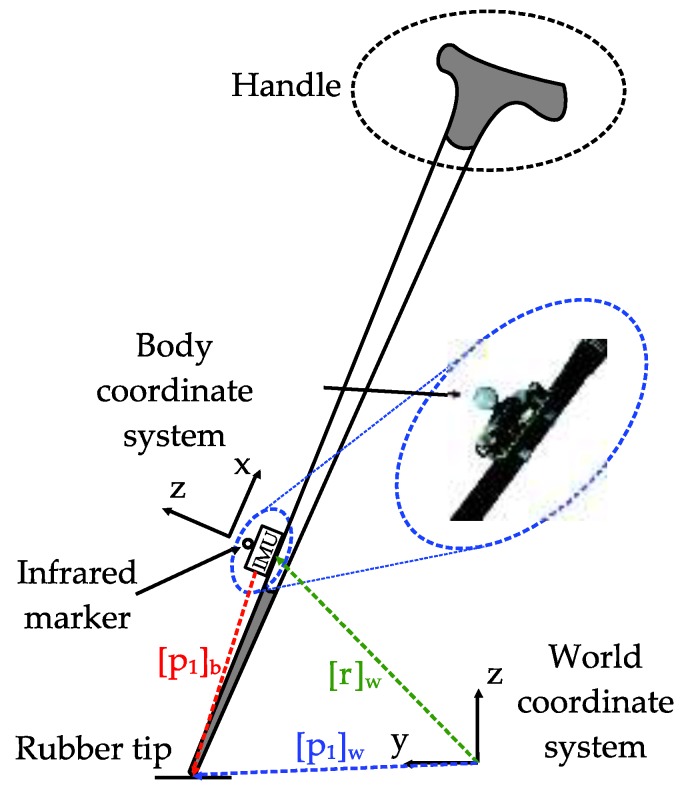
Standard cane with an attached inertial sensor and infrared marker.

The proposed system consists of an inertial sensor unit that is firmly attached to the body of a standard aluminum cane (see Figure 2). The cane is equipped with a rubber tip and a handle on top to minimize the shock when walking. An Xsens MTi1, which is an inertial measurement unit (IMU), is mounted on a customized board with Bluetooth communication and SD card storage. The IMU has a three-axis accelerometer, three-axis gyroscope, and three-axis magnetometer. To provide reference measurements, there is an infrared marker for an optical tracker system is put on the surface of the IMU and coincides with the origin of the sensor module. 

We use two additional duplicate measurement units attached to different positions on the standard cane, and one additional unit on a quadripod cane for performance analysis.

Two coordinate systems are used: the body coordinate system {*B*} associated with the IMU and the world coordinate system {*W*}, which is a fixed reference coordinate system. It is assumed that the *z*-axis of the world coordinate frame is parallel to the direction of local gravitational acceleration and pointing upward. The *y*-axis of this frame is chosen to point in the walking direction. The three axes of the body coordinate system coincide with the three axes of the inertial sensor (see Figure 2).

Let [*p*_1_]*_b_* ∈ ℝ^3^ and [*r*]*_w_* ∈ ℝ^3^ be 3-D vectors that represent the position of the tip of the cane and the inertial sensor (and the infrared marker), respectively. The subscript *b* is used to denote the representation in the body coordinate system. Similarly, subscript *w* is used for the world coordinate system.

## 3. Methodology

The main objective of this work is to estimate walking distances from sensory information. There are basically three approaches for walking distance estimation using sensors mounted on the foot, waist, or wrist. The indirect method counts the number of strides traveled and the time interval between each two consecutive strides (or one step interval). Assuming that the user performs continuous walking with small variation in speed, the walking distance can be estimated using a linear relationship between the walking step length and the walking rate [21,22]. This algorithm is very simple, but the accuracy is not guaranteed due to the various assumptions.

The second method is based on successive double-integral-based step length estimation from acceleration. The major drawback of this technique is that error rapidly accumulates over the integration time. However, this problem can be partially solved by using a measurement update scheme called zero-velocity updates (ZUPT), where the estimation error can be compensated or reset during the zero-velocity intervals [23].

Another recent method applies a verified relationship between the vertical acceleration and step length to obtain the walking distance by summing all step lengths during travel [24]. However, this method also relies on the assumption of continuous walking and normal walking speed.

In this study, the movement of the measurement unit on a cane is different from cases where a sensor module is attached to a human body. Thus, we used the double integration method to estimate the step length with measurement updating in a Kalman filter based on the ZUPT method. The walking distance is then calculated by summing the total walking step lengths. Recent research has focused on estimating the position in 2D or 3D, but our goal is estimating the walking distance and *z*-axis position since there is no heading correction method in our algorithm.

### 3.1. Cane Movement and Walking Phase Detection

The movements of different types of canes during walking are not the same. A quadripod cane’s movement is dominated by translation movement, while a standard cane’s movement is more similar to that of the human foot. A quadripod cane has four legs that are in contact with the ground for a long period. Therefore, there are zero-velocity intervals and they do not depend on the location of the sensor on the cane. However, a standard cane has only one point that is in contact with the ground. If the sensor unit is mounted exactly at the contact point, we can detect the zero-velocity intervals during walking by using similar detection methods to those used for foot-mounted sensors. However, it is virtually impossible to install a sensor at this position. In this section, we compare the two types of canes in terms of how they move and analyze the output of sensors attached to them.

There are many ways to classify the current state of the cane using data from inertial sensor outputs. One simple solution is feature extraction based on the norms of gyroscope and accelerometer output, which is similar to gait phase classification [22,23]. These states and the transitions between them are sometimes not clearly distinguished due to unexpected user behaviors. However, this can be neglected by using a machine learning approach with additional data from other types of sensor sources such as a force sensor [10], but this requires more computational power. In our work, we used a simple algorithm based on a set of thresholds on different quantities of sensor outputs with some additional constraints to classify different phases of the walking action with canes (see Section 3.3).

#### 3.1.1. Quadripod Cane

**Figure 3:**
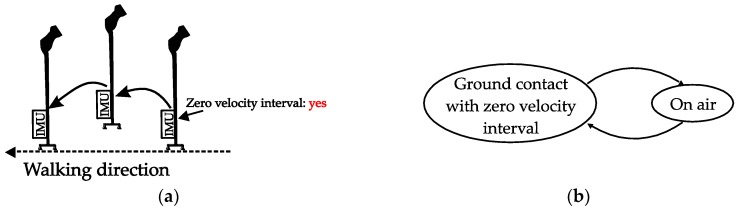
Quadripod cane movement: (**a**) Visualization; (**b**) State diagram.

As shown in Figure 3, the movement of a quadripod cane during walking can be separated into two phases:

1. Ground contact (with zero-velocity interval): All four legs are on the ground and the cane is not moving. There is no rotation of the sensor unit during the ground contact.
2. On air: The legs are not on the ground and the cane is freely moving.

**Figure 4:**
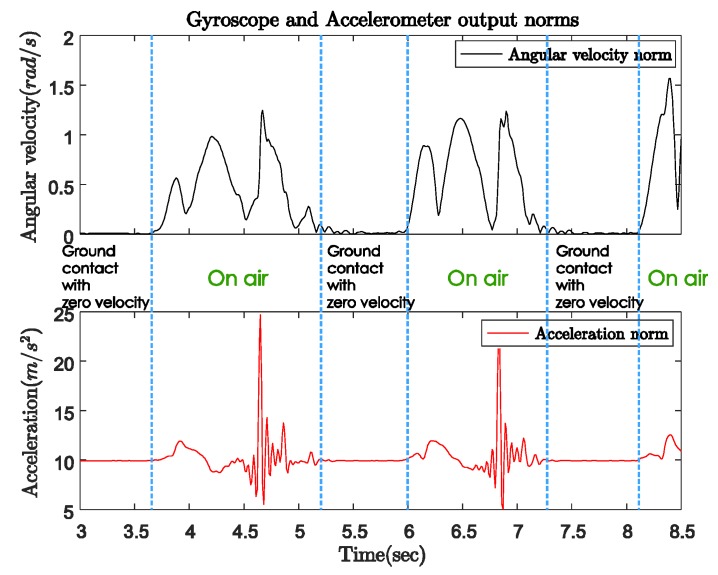
Gyroscope and accelerometer output norms with quadripod cane.

Figure 4 shows the norms of the gyroscope and accelerometer output during normal walking with a quadripod cane. In this case, the sensor unit can be attached anywhere on the body of the cane. The cane is left standing by itself before the user starts walking. We can see the same pattern of sensor output norms in both the periods before walking (from 3 to 3.6 s) and during ground contact (from 5.2 to 6 s) while walking. In these intervals, the angular velocity norm is almost zero, which means there is no rotation movement. The acceleration norm is approximately equal to gravitational acceleration since there is only sensor noise but no movement. As the cane takes off from the ground until the next moment of ground contact (on air period), both the gyroscope and accelerometer outputs have larger positive norms.

**Figure 5:**
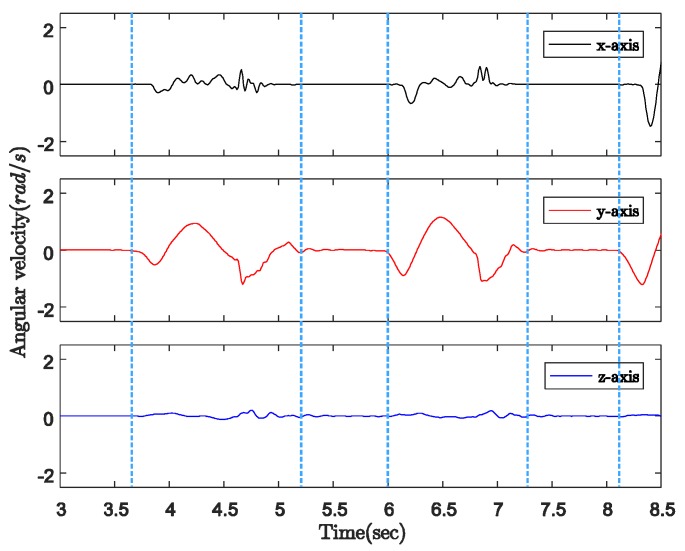
Three-axis angular velocity from gyroscope output with quadripod cane.

**Figure 6:**
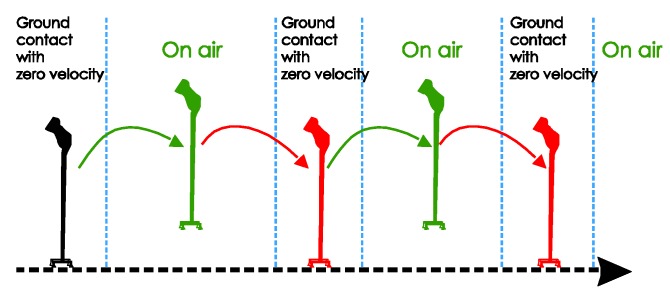
Quadripod cane’s movement patterns.

Figure 5 indicates the three-axis angular velocity corresponding to that of the sensor output in Figure 4. The angular velocity is dominated by a fixed-axis rotation (*y*-axis). During the ground contact period, the gyroscope outputs along all three axes are zero with some small sensor noise. Therefore, as shown in Figure 6, we can detect the walking phases with a quadripod cane based on the accelerometer and gyroscope outputs as follows:

- Ground contact (with zero-velocity interval): No external acceleration and no angular velocity, only affected by gravitational acceleration.
- On air: Contains the contact shock moment, and acceleration and angular velocity are significant.

#### 3.1.2. Standard cane

**Figure 7:**
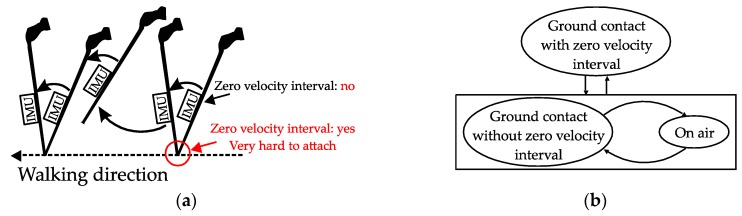
Standard cane movement: (**a**) Visualization; (**b**) State diagram.

The movement of the standard cane while walking can be divided into three phases, as shown in Figure 7:

1. Ground contact with zero-velocity interval: the cane’s tip is on the ground and the cane is not moving or swinging.
2. On air (swing): the cane’s tip is not on the ground and the cane is freely moving.
3. Ground contact without zero-velocity interval: the cane’s tip is on the ground and the cane movement can be modeled as an inverted pendulum.

**Figure 8:**
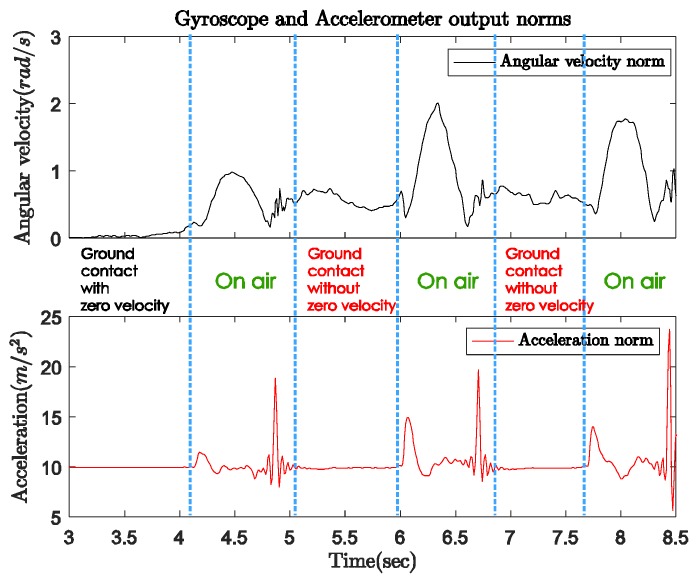
Gyroscope and accelerometer output norms with standard cane.

**Figure 9:**
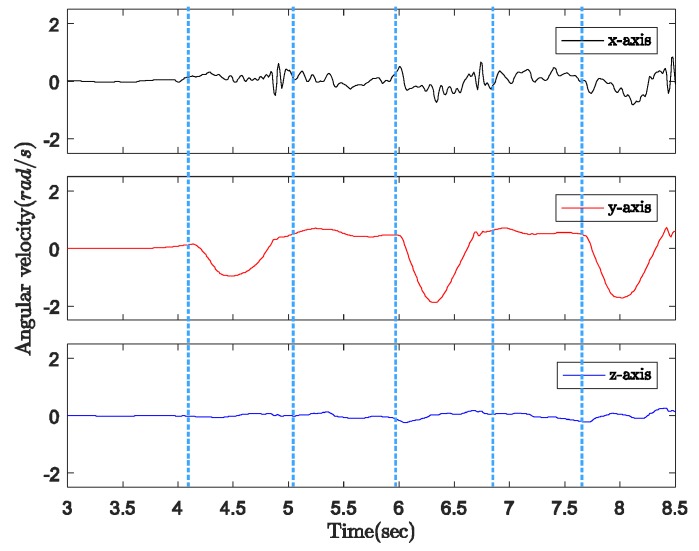
Three-axis angular velocity from gyroscope output with standard cane.

**Figure 10:**
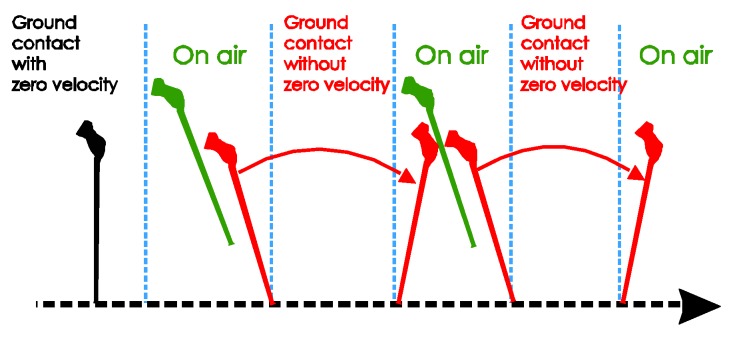
Standard cane’s movement patterns.

Figure 8 shows the norms of the gyroscope and accelerometer output during normal walking with a standard cane. In this case, the sensor unit can be attached anywhere near the tip of the cane. The user was standing still with the cane straight down to the ground before starting walking. Figure 9 shows the three-axis angular velocity corresponding to that of the sensor outputs in Figure 8. The experiments revealed a typical pattern, as shown in Figure 10.

In the beginning when the cane is not moving, the angular velocity norm is almost zero, and the acceleration norm is equal to gravitational acceleration since there is no movement. As the cane takes off from the ground, both the gyroscope and accelerometer outputs have larger positive norms. Lastly, from the moment of contact until the cane takes off again, the dominant movement is rotation. Therefore, the angular velocity norm still has a large positive value when the acceleration norm is approximately constant near the gravitational acceleration (see Figure 8).

In Figure 9, the angular velocity is dominated by a fixed axis rotation (*y*-axis) when the sensor module is attached to the body of the cane. During ground contact periods with or without zero-velocity intervals, the gyroscope outputs along the *y*-axis are assumed to be constant at different levels. Therefore, we can briefly describe the walking phases as follows:

- Ground contact with zero-velocity interval: No external acceleration and no angular velocity.
- Ground contact without zero-velocity interval: Occurs after contact shock moment, very small external acceleration, angular velocity can be assumed as constant.
- On air (swing): Before contact shock moment, acceleration and angular velocity are significant.

### 3.2. Standard Inertial Navigation Using Indirect Kalman Filter

In this section, the positions of the cane’s tip and the sensor are estimated using an inertial navigation algorithm, which was used in a previous study [23]. The indirect Kalman filter is applied with measurement update during ground contact time (see Section 3.4).

The inertial sensor unit’s output data consists accelerometer output *y_a_* ∈ ℝ^3^ and gyroscope output *y_g_* ∈ ℝ^3^, which are represented in the body coordinate system as:

(1) $$\begin{array}{l} y_{a}={\left[a\right]}_{b}+{\left(C_{b}^{w}\right)}^{T}{\left[\tilde{g}\right]}_{w}+u_{a}+b_{a}\\ y_{g}={\left[\omega \right]}_{b}+u_{g}+b_{g}, \end{array}$$

where $C_{b}^{w}\in \mathrm{SO}\left(3\right)$ represents a rotation from the body (sensor) coordinate system to the world coordinate system; *a* ∈ ℝ^3^ is the acceleration produced by force other than the gravitational field; *ω* ∈ ℝ^3^ is the body angular rate; and *b_a_*,*b_g_* ∈ ℝ^3^ are the accelerometer and gyroscope bias, respectively. $\tilde{g}$ is defined by:

$$\tilde{g}≜\left[\begin{array}{c} 0\\ 0\\ g \end{array}\right],$$

where *g* is the gravitational acceleration. The sensor noise *u_a_*,*u_g_* ∈ ℝ^3^ are assumed to be zero-mean white Gaussian noise satisfying:

$$\begin{array}{l} E\left\{u_{a}\left(t\right)u_{a}{\left(s\right)}^{T}\right\}=r_{a}I\delta \left(t-s\right)\\ E\left\{u_{g}\left(t\right)u_{g}{\left(s\right)}^{T}\right\}=r_{g}I\delta \left(t-s\right). \end{array}$$

A quaternion *q* ∈ ℝ^4^ is used to represent the rotation between the world and body coordinate systems. It is related to rotation matrix $C_{b}^{w}$ as:

$$C\left(q\right)={\left(C_{b}^{w}\right)}^{T}.$$

Let *r* = [*r*]*_w_* ∈ ℝ^3^ and *v* = [*v*]*_w_* ∈ ℝ^3^ be the position and velocity of the sensor in the world coordinate system. The basic equations for inertial navigation are:

(2) $$\begin{array}{l} \dot{q}=\frac{1}{2}\Omega \left(\omega \right)q\\ \dot{v}={\left[a\right]}_{w}=C^{T}\left(q\right){\left[a\right]}_{b}\\ \dot{r}=v, \end{array}$$

where Ω is defined by:

$$\Omega \left(\omega \right)≜\left[\begin{array}{cccc} 0 & -\omega_{x} & -\omega_{y} & -\omega_{z}\\ \omega_{x} & 0 & \omega_{z} & -\omega_{y}\\ \omega_{y} & -\omega_{z} & 0 & \omega_{x}\\ \omega_{z} & \omega_{y} & -\omega_{x} & 0 \end{array}\right].$$

Since the position and velocity can be derived from sensor data using (1) and (2), we can use either a direct Kalman filter or indirect Kalman filter for the navigation algorithm. In a direct Kalman filter, *r* and *q* are included in the state and are directly estimated in the filter. In an indirect Kalman filter, *r* and *q* are computed using (2), where *y_g_* and $y_{a}-C\left(q\right)\tilde{g}$ are used in place of *ω* and [*a*]*_b_*, respectively. Since *y_g_* ≠ *ω* and $y_{a}-C\left(q\right)\tilde{g}\neq a_{b}$ due to the noise in (1), the computed values contain some error, which is estimated in an indirect Kalman filter. Among two type of filters, it seems that the indirect Kalman filter is more popular since the number of state is smaller and there is no required assumption for the derivative of *ω* [25]. In this paper, a quaternion-based indirect Kalman filter is used for both the standard algorithm and the proposed algorithm.

Let $\hat{q},\hat{v},\hat{r}$ be the computed values of *q,v,r,* using (2):

(3) $$\begin{array}{l} \dot{\hat{q}}=\frac{1}{2}\Omega \left(y_{g}\right)\hat{q}\\ \dot{\hat{v}}=C^{T}\left(\hat{q}\right)y_{a}-\tilde{g}\\ \dot{\hat{r}}=\hat{v}. \end{array}$$

Due to the measurement noise *u_g_* and *u_a_*, there is error in the estimation of $\hat{q},\hat{v},\hat{r}$. The errors in $\hat{v}$ and $\hat{r}$ are *v_e_* and *r_e_*, defined by:

(4) $$\begin{array}{l} v_{e}≜v-\hat{v}\\ r_{e}≜r-\hat{r}. \end{array}$$

The error in $\hat{q}$ can be expressed using the multiplicative quaternion error. The quaternion error ${\bar{q}}_{e}\in \;{\mathbb{R}}^{4}$ satisfies the following expression:

(5) $$q=\hat{q}⊗{\bar{q}}_{e},$$

or the equivalent representation:

(6) $$C\left(q\right)=C\left({\bar{q}}_{e}\right)C\left(\hat{q}\right),$$

where ⊗ is the quaternion multiplication operator. We assume that ${\bar{q}}_{e}$ is small and can be approximated by:

(7) $${\bar{q}}_{e}\approx \left[\begin{array}{c} 1\\ q_{e} \end{array}\right].$$

We can also approximate $C({\bar{q}}_{e})$ when *q_e_* is small using the following equation:

(8) $$C\left({\bar{q}}_{e}\right)\approx I-2\left[q_{e}\times \right],$$

where $\left[p\times \right]\left(p={\left[\begin{array}{ccc} p_{1} & p_{2} & p_{3} \end{array}\right]}^{T}\in {\mathbb{R}}^{3}\right)$ is defined by:

$$\left[p\times \right]≜\left[\begin{array}{ccc} 0 & -p_{3} & p_{2}\\ p_{3} & 0 & -p_{1}\\ -p_{2} & p_{1} & 0 \end{array}\right].$$

The state variable of an indirect Kalman filter are defined by:

(9) $$x_{std}≜\left[\begin{array}{c} q_{e}\\ r_{e}\\ v_{e} \end{array}\right]\in {\mathbb{R}}^{9\times 1}.$$

The state equation is:

(10) $${\dot{x}}_{std}=\left[\begin{array}{ccc} \left[y_{g}\times \right] & 0 & 0\\ 0 & 0 & I_{3}\\ -2C^{T}\left(\hat{q}\right)\left[y_{a}\times \right] & 0 & 0 \end{array}\right]x_{std}+\left[\begin{array}{c} -\frac{1}{2}u_{g}\\ 0\\ -C^{T}\left(\hat{q}\right)u_{a} \end{array}\right],$$

where the covariance matrix is:

$$E\left\{\left[\begin{array}{c} -\frac{1}{2}u_{g}\\ 0\\ -C^{T}\left(\hat{q}\right)u_{a} \end{array}\right]{\left[\begin{array}{c} -\frac{1}{2}u_{g}\\ 0\\ -C^{T}\left(\hat{q}\right)u_{a} \end{array}\right]}^{T}\right\}=\left[\begin{array}{ccc} 0.25R_{g} & 0 & 0\\ 0 & 0 & 0\\ 0 & 0 & C^{T}\left(\hat{q}\right)R_{a}C\left(\hat{q}\right) \end{array}\right].$$

### 3.3. Walking Phase Classification

The first step to estimate the walking distance is to detect whether a person is walking or not. During walking, the movement is constrained (the cane rotation axis is constrained), and the angle between the cane and vertical direction are within a certain range.

For the movement constraint, the cane rotations are dominated by vertical rotations when changing the walking direction and lateral rotations when the cane swings during walking. This means that the gyroscope signals are mostly concentrated on the *x*-axis and *y*-axis. To check whether this condition is satisfied, signal magnitude subtraction (SMS) of the gyroscope output is introduced, which involves subtracting the *xy*-axis gyroscope signal from the angular velocity norm:

(11) $$SMS=\left\|y_{g}\right\|-\sqrt{y_{g,x}^{2}+y_{g,y}^{2}},$$

where $y_{g,x}$ and $y_{g,y}$ are the angular velocity along the *x*-axis and *y*-axis of the sensor coordinate frame, and $\left\|y_{g}\right\|=\sqrt{y_{g,x}^{2}+y_{g,y}^{2}+y_{g,z}^{2}}$ is the angular velocity norm.

The discrete time *k* is assumed to be part of either the walking interval or the stationary interval if the following condition is satisfied:

(12) $${\bar{SMS}}_{k}\leq B_{sms},\;{\bar{SMS}}_{k}=\left(\sum_{i=k-N_{sms}/2}^{k+N_{sms}/2}SMS_{i}\right)/N_{sms},$$

where *B_sms_* is a threshold and *N_sms_* is an integer. Otherwise, k is part of non-walking periods.

For the cane angle condition, the *x*-axis of the sensor coordinate frame (Figure 2) and the upward vertical direction make an acute angle during walking. The cane’s angle is calculated from the acceleration signal:

(13) $$\theta =\left|\tan^{-1}\left(\frac{\sqrt{y_{a,y}^{2}+y_{a,z}^{2}}}{y_{a,x}}\right)\right|,$$

where $y_{a,x},y_{a,y}$ and $y_{a,z}$ are the acceleration along the three axes of the sensor coordinate frame.

The cane is assumed to point downwards if the following condition is satisfied:

(14) $${\bar{\theta }}_{k}\leq B_{theta},\;{\bar{\theta }}_{k}=\left(\sum_{i=k-N_{theta}/2}^{k+N_{theta}/2}\theta_{i}\right)/N_{theta}.$$

The proposed algorithm detects walking movement based on the following criteria:

- First criterion: a walking interval is detected using (12) and (14).
- Second criterion: the duration satisfies:
  (15) $$\Delta k=k_{s}-k_{e}>K,$$

where *k_s_* and *k_e_* denote the start and end points of a walking interval (detected by (12) and (14)).

An experiment was performed to assess the performance of the proposed method. A subject was asked to use the standard cane and perform the following actions:

1. The cane is lying on the table
2. Lifting the cane up and doing some random waving actions
3. Leaning the cane on the table
4. Lifting and holding the cane against the ground and preparing to walk
5. Walking along a straight line
6. Stopping and turning around
7. Standing still and doing some waving actions
8. Stopping waving and starting to walk freely
9. Stopping walking and doing some waving actions
10. Stopping waving and leaning the cane on the table
11. Lifting the cane up and putting it on a table.

**Figure 11:**
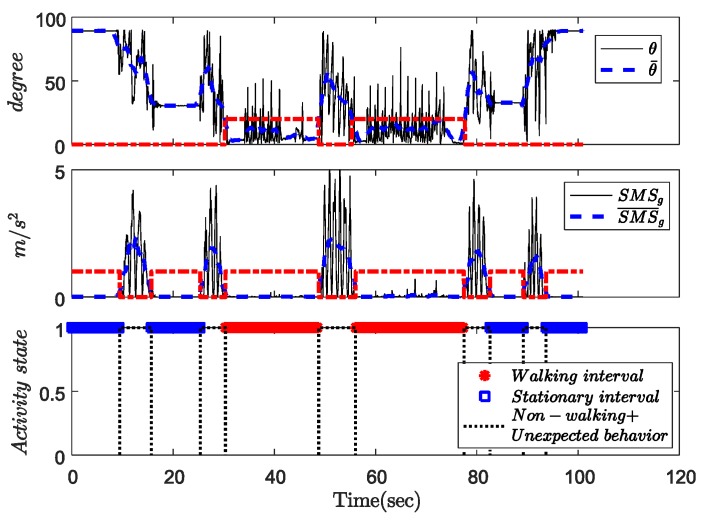
An example of activity classification. Upper graph: Cane angle *θ*. Middle graph: SMS signal. Bottom graph: Activity classification result.

The walking interval detection results are shown in Figure 11. The upper and middle graphs show the calculated cane angle, the SMS signal, and the condition outputs (red lines). The activity classification results are shown in the bottom graph. The results show that the actions 1, 3, 10, and 11 contain stationary intervals. The actions 2, 4, 7 and 9 contain non-walking intervals and unexpected behaviors. The remaining periods, including actions 5, 6, and 8 are assigned as walking intervals. The results show that the proposed algorithm can classify the walking movements correctly.

After detecting walking periods, we need to classify the walking phases and update the measurements to eliminate the estimation error. For both the quadripod cane and standard cane, we use similar methods to detect the ground contact phases and swing phases (on air) based on the accelerometer and gyroscope output.

For each sampling time *k*, we evaluate the following conditions:

(16) $$\left|g-\left\|y_{a,i}\right\|\right|\leq B_{a},\;k-N_{a}/2\leq i\leq k+N_{a}/2,$$

(17) $$\left\|y_{g,i}\right\|\leq B_{g},\;\;k-N_{g}/2\leq i\leq k+N_{g}/2,$$

where *g* is standard gravity, the parameters *B_a_* and *B_g_* are thresholds, *N_a_* and *N_g_* specify time intervals.

**Figure 12:**
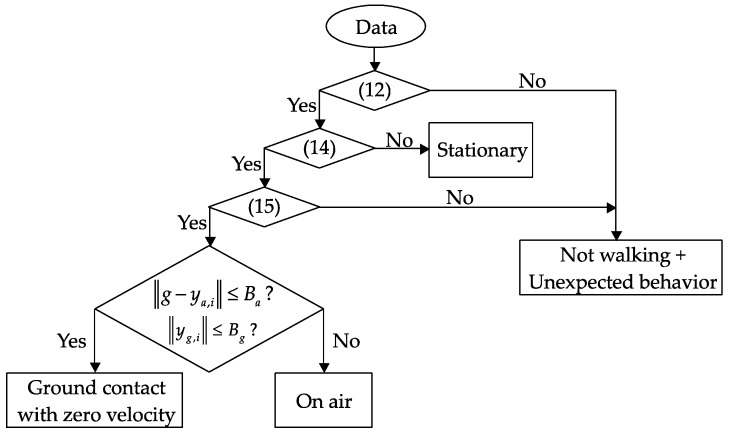
Flowchart of walking phase classification for a quadripod cane.

In the case of quadripod cane, the zero-velocity intervals coincide with the ground contact intervals and are detected if there is no external acceleration and no angular velocity. Therefore, at any time, if the angular velocity norm is smaller than one threshold value and the acceleration norm is near the standard gravity for more than a specified time (both (16) and (17) are satisfied), it can be assigned as ”ground contact with zero velocity”. The remaining periods after all of ground contact periods are detected are assigned as “on air” or swing phases. Figure 12 shows a flowchart of the walking phase classification algorithm for the quadripod cane.

**Figure 13:**
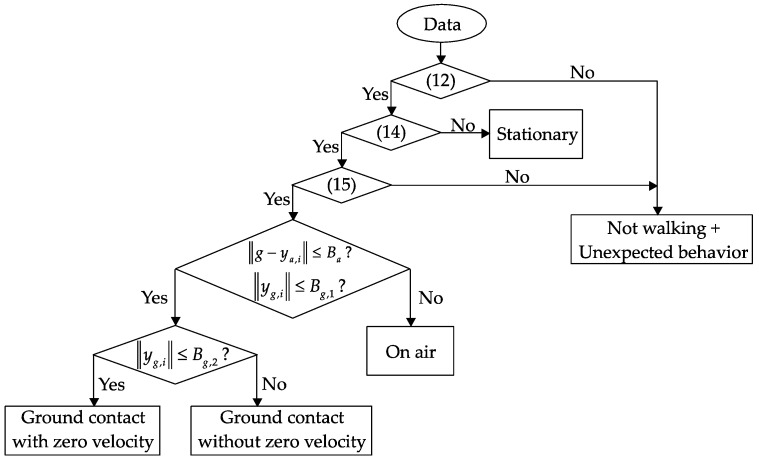
Flowchart of walking phase classification for a standard cane.

With the standard cane, the detection is different since the zero-velocity intervals do not always coincide with the ground contact phases. The ground contact phases in this case (with or without zero-velocity intervals) can be separated from swing phases using similar conditions to those applied for the quadripod cane (see Figure 13). The intervals of ground contact with zero velocity, which occur before and after walking, can then be detected with a stronger condition for the angular velocity norm. This is understandable since such intervals occur during walking with very small external acceleration, and angular velocity is generated due to the inverted-pendulum-like movement of the cane. The remaining periods, which have very small external acceleration and small angular velocity, are assigned as the “ground contact with zero velocity” intervals.

### 3.4. Measurement Updating

For the quadripod cane, the linear velocity of the sensor module is zero during the ground contact phases with zero velocity. Then if both Equations (16) and (17) are satisfied, the measurement update equation is:

(18) $$0-{\hat{v}}_{k}=v_{e}+u_{k},$$

where *u_k_* is the measurement noise:

$$E\left\{u_{k}u_{k}^{T}\right\}=r_{u}I_{3}.$$

**Figure 14:**
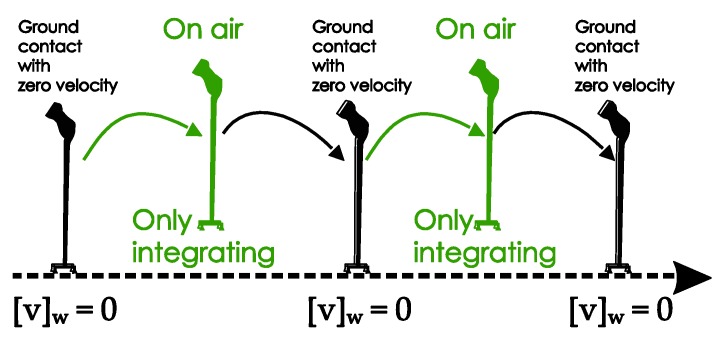
Velocity updating procedures for a quadripod cane.

The update procedure is shown in Figure 14:

For the standard cane, we do not have the actual zero velocity of the sensor module since the cane is moving during the ground contact phases of walking. However, the tip of the cane, which is the only contact point, is not moving in those periods. The velocity of the sensor unit can be estimated using the inverted pendulum model.

The position of the cane’s tip in the world coordinate system (see Figure 2) is:

(19) $${\left[p_{1}\right]}_{w}={\left[r\right]}_{w}+C_{b}^{w}{\left[p_{1}\right]}_{b}.$$

When the tip is on the ground, ${\left[p_{1}\right]}_{w}$ is constant. Therefore, taking the derivative of (19) leads to:

(20) $$0={\left[{\dot{p}}_{1}\right]}_{w}={\left[\dot{r}\right]}_{w}+{\dot{C}}_{b}^{w}{\left[p_{1}\right]}_{b}.$$

Using the following property:

$${\dot{C}}_{b}^{w}=-C_{b}^{w}{\left[\omega \times \right]}^{T}=C_{b}^{w}\left[\omega \times \right],$$

we have:

(21) $$0={\left[\dot{r}\right]}_{w}+C_{b}^{w}\left[\omega \times \right]{\left[p_{1}\right]}_{b},$$

and the velocity of the sensor unit is:

(22) $${\left[v\right]}_{w}={\left[\dot{r}\right]}_{w}=-C_{b}^{w}\left[\omega \times \right]{\left[p_{1}\right]}_{b}=C_{b}^{w}\left[{\left[p_{1}\right]}_{b}\times \right]\omega .$$

Before and after the walking, the cane’s tip is on the ground and the cane is not moving, and both conditions (16) and (17) are satisfied. The corresponding velocity of the inertial sensor unit is zero. The measurement update equation is:

(23) $$0-{\hat{v}}_{k}=v_{e}+u_{1,k},$$

where $u_{1,k}$ is the measurement noise

$$E\left\{u_{1,k}u_{1,k}^{T}\right\}=r_{u,1}I_{3}.$$

If only (16) is satisfied at discrete time *k*, then there is no zero-velocity interval. Hence, the cane is moving as an inverted pendulum while the tip is on the ground. The corresponding velocity of the sensor unit is updated using (22) as follows:

(24) $$C{\left({\hat{q}}_{k}\right)}^{T}\left[{\left[p_{1}\right]}_{b}\times \right]y_{g,k}-{\hat{v}}_{k}=v_{e}+u_{2,k},$$

where $u_{2,k}$ is also the measurement noise:

$$E\left\{u_{2,k}u_{2,k}^{T}\right\}=r_{u,2}I_{3}.$$

**Figure 15:**
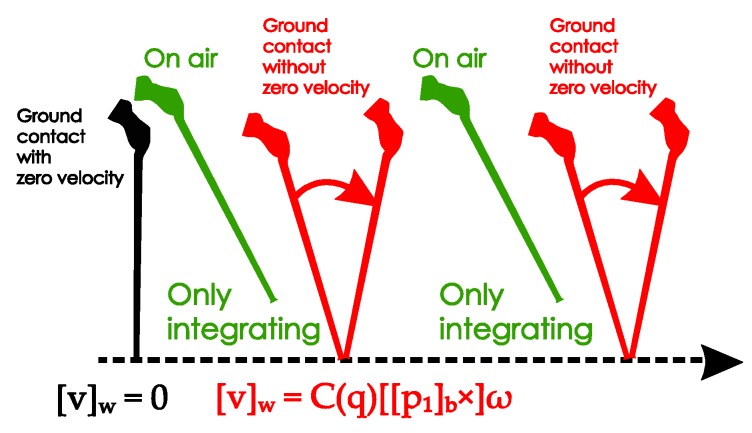
Velocity updating procedures for a standard cane.

The update procedure is shown in Figure 15:

### 3.5. Walking Distance Estimation Based on Measurement Update

With the quadripod cane, the external acceleration and angular velocity affecting the sensor unit are almost zero during ground contact since there is no linear or rotational movement. Hence, we applied the standard inertial navigation algorithm (Section 3.2) using Equation (3) to estimate the position and velocity of the sensor module. The walking distance is then calculated by summing the walking step lengths between two consecutive ground contact moments.

Let *S_j_* and *E_j_* be the starting and ending indices of the *j*-th walking step. The step length *SL_j_* and walking distance *WL* can be computed as follows:

(25) $$SL_{j}=\left\|r_{E_{j}}-r_{S_{j}}\right\|,$$

(26) $$WL=\sum_{1}^{n}SL_{j}.$$

For the standard cane, we need to investigate the effect of the swing motion to reduce the error of the estimation. Taking the derivative of (22) leads to:

(27) $$\begin{array}{l} {\left[a\right]}_{w}={\left[\dot{v}\right]}_{w}={\dot{C}}_{b}^{w}\left[{\left[p_{1}\right]}_{b}\times \right]\omega +C_{b}^{w}\left[{\left[p_{1}\right]}_{b}\times \right]\dot{\omega }\\ =C_{b}^{w}\left[\omega \times \right]\left[{\left[p_{1}\right]}_{b}\times \right]\omega +C_{b}^{w}\left[{\left[p_{1}\right]}_{b}\times \right]\dot{\omega }\\ =C_{b}^{w}\left[{\left[p_{1}\right]}_{b}\times \right]\dot{\omega }-C_{b}^{w}\left[{\left[p_{1}\right]}_{b}\times \right]\left[\omega \times \right]\omega \\ =C_{b}^{w}\left[{\left[p_{1}\right]}_{b}\times \right]\dot{\omega }. \end{array}$$

Therefore, the accelerometer output $y_{a}$ can be modeled as:

(28) $$y_{a}={\left(C_{b}^{w}\right)}^{T}{\left[\tilde{g}\right]}_{w}+\left[{\left[p_{1}\right]}_{b}\times \right]\dot{\omega }.$$

**Figure 16:**
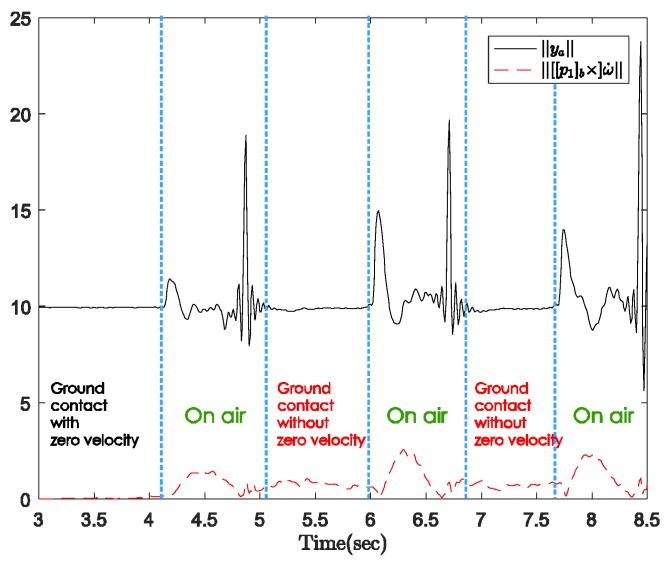
Acceleration norm and external acceleration.

The acceleration *y_a_* in Equation (28) is only gravitational acceleration if the angular velocity *ω* is constant. In other words, the external acceleration is very small and can be neglected in the ground contact periods (with or without zero velocity) based on the assumption that the angular velocity of the sensor unit is constant during the swing phases. We plotted data from the sensor outputs (Figure 16) and compared the acceleration norm with the external acceleration (the second part in Equation (28)). In the intervals mentioned above, the external acceleration caused by movement is significantly smaller than the gravitational acceleration. Therefore, the assumption is acceptable, and the standard inertial navigation algorithm can still be used to estimate the sensor unit’s velocity and position.

In a scenario where the ground is flat and has zero slope, we can also include the *z*-axis position of the tip in the state variable of the Kalman filter as a measurement that is updated using (6), (8), and (19) in the following estimation:

(29) $${\left[p_{1}\right]}_{w}={\left[r\right]}_{w}+C_{b}^{w}\left(\hat{q}\right)\left(I+2\left[\bar{q}\times \right]\right){\left[p_{1}\right]}_{b}$$

(30) $${\left[p_{1}\right]}_{w}-{\left[\hat{r}\right]}_{w}-C_{b}^{w}\left(\hat{q}\right){\left[p_{1}\right]}_{b}=r_{e}-2C_{b}^{w}\left(\hat{q}\right)\left[{\left[p_{1}\right]}_{b}\times \right]\bar{q}.$$

## 4. Experiments and Results

**Figure 17:**
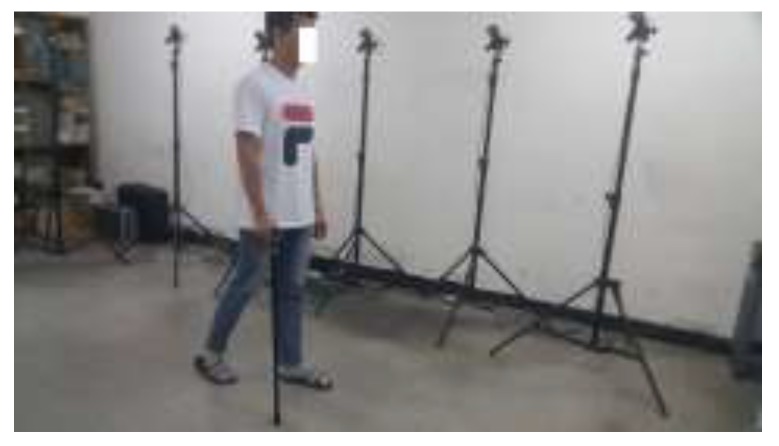
Continuous walking within the working range of an optical tracker system.

Experiment results are presented to verify the proposed method. In the first experiment, a subject walked 10 times with the quadripod cane along a 3-m path within the working range of an optical tracker system (see Figure 17). The sensor module and an infrared marker are attached at the same place on the body of the quadripod cane. Both the inertial sensors and camera system are configured to have output data at 100 Hz.

**Figure 18:**
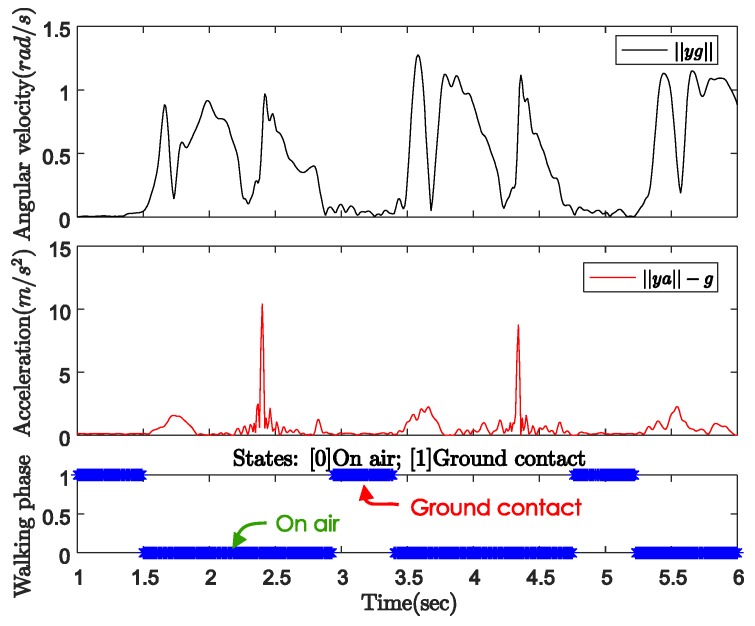
Quadripod cane’s output data and zero-velocity intervals.

One of the results is given in Figure 18 with the sensor outputs and zero-velocity intervals, which are detected using (16) and (17) with the following parameters:

(31) $$\begin{array}{l} B_{g}=0.4,B_{a}=0.3,N_{g}=N_{a}=15,B_{sms}=0.2,N_{sms}=200,B_{theta}=20,N_{sms}=200,\\ r_{g}=0.0001,r_{a}=0.0005,r_{u,1}=r_{u,2}=0.001. \end{array}$$

Table 1 shows the estimated walking distances using standard inertial navigation algorithm with zero-velocity updating and the reference values obtained from the optical tracker system. During zero-velocity intervals, there is no movement of the quadripod cane, so the angular velocity is very small (almost zero) and the acceleration is near gravitational acceleration. The average absolute error is about 9.8 cm, while the maximum error is about 13.4 cm.

In the second experiment, a similar procedure to the first experiment is repeated with the standard cane. Three sensor modules and three infrared markers are firmly attached at three different positions (32) on the body of the standard cane. The parameters used in the algorithm are:

(32) $${\left[p_{1}\right]}_{b}\in \left\{{\left[\begin{array}{ccc} -0.315 & 0 & -0.017 \end{array}\right]}^{T};{\left[\begin{array}{ccc} -0.575 & 0 & -0.017 \end{array}\right]}^{T};{\left[\begin{array}{ccc} -0.778 & 0 & -0.016 \end{array}\right]}^{T}\right\}$$

(33) $$\begin{array}{l} B_{g,1}=0.3,B_{g,2}=0.2,B_{a}=0.2,N_{g}=20,N_{a}=25,\\ B_{sms}=0.2,N_{sms}=200,B_{theta}=20,N_{sms}=200,\\ r_{g}=0.0001,r_{a}=0.0005,r_{u,1}=r_{u,2}=0.001. \end{array}$$

**Figure 19:**
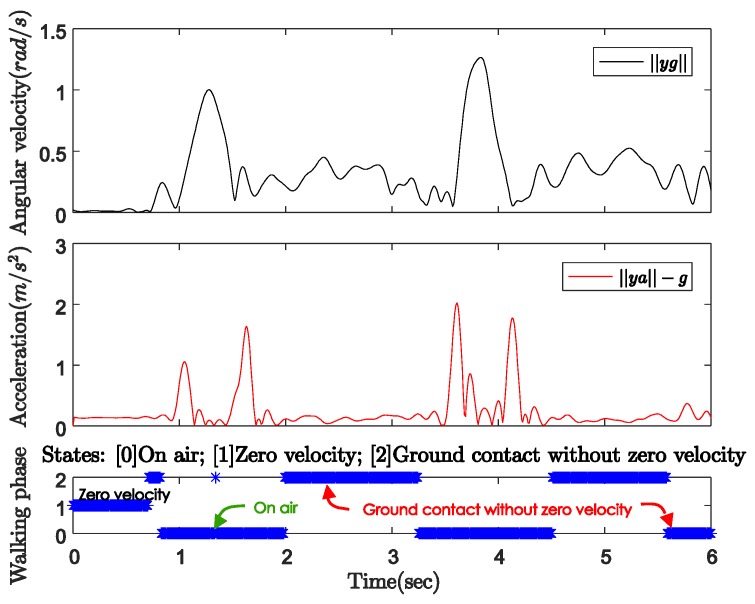
Standard cane’s output data and activity classification.

One of the results with sensor output norms and walking phase classification results is given in Figure 19.

Figure 19 shows that zero-velocity intervals occur only right before and after walking, which means the standard cane moves like an inverted pendulum during the whole ground contact phase. The gyroscope’s output norm during ground contact without zero velocity is smaller than that during the swing (on air) phase but not near zero anymore. Table 2 presents the results for the three different positions of the sensor modules. The results are quite good with a mean absolute errors for each case of about 5.0, 6.2, and 18.6 cm. The closer the sensor module is to the contact point; the better estimated results are. This is not surprising since the acceleration caused by the rotational movement during the ground contact phase is reduced when the sensor module is attached near the contact point.

A third set of experiments was performed to verify the accuracy of our algorithm for long-distance walking with both types of canes. One sensor module is mounted on each cane at the same position as in the previous experiments, and the standard cane has the sensor module at the lowest position [*p*_1_]*_b_*. An additional sensor module is attached to the right foot of the user. Five people walked five times along a total of 170 m in a flat corridor with zero slope. Each time, the participant walked along 85 m, turned around, and came back to the starting position. Similar parameters to the second experiment were chosen. The method for updating the *z*-axis position in Section 3.5 was also used to verify the performance of the algorithm, and the results are given in Table 3 and Table 4.

**Figure 20:**
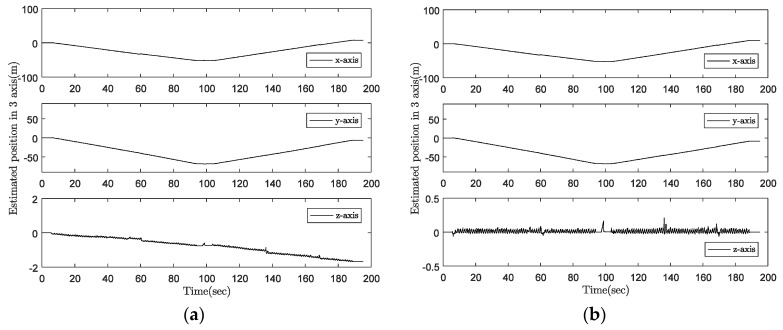
*z*-axis position estimation: (**a**) without *z*-axis updating; (**b**) with *z*-axis updating.

The estimated results of our algorithm are compared with the walking distance calculated from the foot-mounted sensor data using the standard inertial navigation algorithm. One estimated position with two updating methods is given in Figure 20. The result is quite good, and the maximum error for the quadripod cane is 114.2 cm (0.67%) and for the standard cane is 215.4 cm (1.27%). The maximum error of the foot-mounted sensor is 271.6 cm (1.60%).Figure 20 shows that the error in the *z*-axis position of the tip is corrected using our method, which included Equations (29) and (30) in the Kalman filter measurement update. However, there is a little improvement in the *xy* position since the movement range of the *z*-axis is not significant (about 10 cm).

## 5. Conclusions

We have proposed a walking cane device consisting of an inertial sensor and a method for walking phase classification and velocity updating. The system can accurately estimate walking distance when using two types of canes. We can use the standard inertial navigation algorithm with zero-velocity updates for the quadripod cane, but the standard cane’s results show that there is no zero velocity when walking since the moves like an inverted pendulum during the ground contact phase. The maximum estimation error for 170 m of walking was 114.2 cm (0.67%) for the quadripod cane and 215.4 cm (1.27%) for the standard cane. 

One drawback of the proposed algorithm is that it does not work in case of dragging and other abnormal situations since there is no measurement updating interval. In these cases, the error will quickly diverge. The proposed system provides a solution for data collection and analysis, which could be helpful for caregivers or doctors during patient rehabilitation.

## Figures and Tables

**Table 1:** Quadripod cane’s estimated and reference walking distance.

No.	Estimated Length (m)	Optical Tracker (m)	Absolute Error (m)
1	3.418	3.286	0.132
2	3.346	3.255	0.091
3	3.322	3.274	0.049
4	3.374	3.292	0.083
5	3.367	3.303	0.064
6	3.380	3.276	0.105
7	3.374	3.281	0.093
8	3.405	3.271	0.134
9	3.155	3.253	0.099
10	3.397	3.270	0.127
**Mean**	**3.354**	**3.276**	**0.098**

**Table 2:** Standard cane’s estimated and reference walking distance.

No.	${\left[p_{1}\right]}_{b}=\left[-0.315;0;-0.017\right]$			${\left[p_{1}\right]}_{b}=\left[-0.575;0;-0.017\right]$			${\left[p_{1}\right]}_{b}=\left[-0.778;0;-0.016\right]$		
	Estimated (m)	Optical Tracker (m)	Abs. Error (m)	Estimated (m)	Optical Tracker (m)	Abs. Error (m)	Estimated (m)	Optical Tracker (m)	Abs. Error (m)
1	2.934	2.848	0.086	2.823	2.785	0.038	2.596	2.790	0.193
2	2.615	2.537	0.077	2.541	2.543	0.002	2.625	2.547	0.078
3	2.908	2.898	0.010	2.596	2.682	0.086	2.700	2.901	0.202
4	2.927	2.877	0.049	2.883	2.839	0.043	2.706	2.908	0.202
5	2.816	2.764	0.052	2.600	2.742	0.142	2.613	2.794	0.181
6	2.678	2.675	0.002	2.758	2.798	0.040	2.489	2.732	0.243
7	2.916	2.834	0.082	2.809	2.897	0.088	2.520	2.754	0.234
8	2.730	2.701	0.029	2.787	2.799	0.013	2.546	2.827	0.281
9	2.753	2.726	0.027	2.806	2.733	0.073	2.615	2.784	0.170
10	2.681	2.763	0.083	2.734	2.832	0.098	2.839	2.767	0.072
**Mean**	**2.796**	**2.762**	**0.050**	**2.733**	**2.765**	**0.062**	**2.625**	**2.780**	**0.186**

**Table 3:** Estimated walking distance from 170-meter experiment with quadripod cane.

Person Index	Foot-Mounted Sensor		Quadripod Cane Sensor	
	Average Estimated Walking Distance (m)	Standard Deviation (m)	Average Estimated Walking Distance (m)	Standard Deviation (m)
1	172.716	1.043	169.679	1.114
2	169.661	0.531	171.142	0.637
3	170.125	1.019	170.297	0.969
4	169.476	1.572	170.140	1.261
5	169.564	0.862	170.538	1.047

**Table 4:** Estimated walking distance from 170-meter experiment with standard cane.

Person Index	Foot-Mounted Sensor		Standard Cane Sensor, without *z*-axis Position Updating		Standard Cane Sensor, with *z*-axis Position Updating	
	Average Estimated Walking Distance (m)	Standard Deviation (m)	Average Estimated Walking Distance (m)	Standard Deviation (m)	Average Estimated Walking Distance (m)	Standard Deviation (m)
1	170.639	2.114	172.154	1.480	172.121	1.496
2	171.679	0.298	170.963	1.425	170.884	1.415
3	169.931	1.085	171.582	2.576	171.390	2.683
4	170.603	1.029	170.435	1.135	170.432	1.032
5	170.563	1.439	170.782	1.781	170.717	1.549
